# The use of mechanical CPR for IHCA during the COVID-19 pandemic as compared to the pre-pandemic period

**DOI:** 10.1186/s13054-024-04841-2

**Published:** 2024-02-27

**Authors:** Conor Crowley, Justin Salciccioli, Yuxiu Lei, Christopher Hansen, Tomoyoshi Tamura, Edy Y. Kim, Ari Moskowitz, the American Heart Association’s Get With The Guidelines®—Resuscitation Investigators, Anne Grossestreuer, Ari Moskowitz, Joseph Ornato, Matthew Churpek, Monique Anderson Starks, Paul Chan, Saket Girotra, Sarah Perman

**Affiliations:** 1 Division of Pulmonary and Critical Care Medicine, Lahey Hospital and Medical Center, Burlington, MA 01805 USA; 2 Tufts University School of Medicine, Boston, MA 02111 USA; 3 Division of Pulmonary and Critical Care Medicine, Department of Medicine, Brigham and Women’s Hospital, Boston, MA 02115 USA; 4 Harvard Medical School, Boston, MA 02115 USA; 5 Department of Emergency and Critical Care Medicine, Keio University School of Medicine, Tokyo, Japan; 6 Division of Critical Care Medicine, Montefiore Medical Center, The Bronx, NY USA

Timely and effective chest compressions are one of the few therapies with clear benefit in cardiac arrest [1]. Mechanical cardiopulmonary resuscitation (mCPR) devices have been developed to provide continuous high-quality CPR and help avoid interruption, compressor fatigue, and variations in compression depth, all of which have been associated poor outcomes [2]. mCPR has largely been investigated in the out of hospital setting with uneven results [3, 4]. In September 2020, the American Heart Association recommended that hospitals consider using mCPR for IHCA during the COVID-19 pandemic to limit healthcare worker exposure to aerosolized virus while providing CPR [5].

Whether the use of mCPR for in-hospital cardiac arrest (IHCA) resuscitation changed in response to COVID-19 has not been explored. We aimed to assess trends in the use of mCPR in relation to the COVID-19 pandemic using the American Heart Association’s (AHA) Get With The Guidelines Database (GWTG).

The GWTG database is a prospective, multicenter registry of IHCA in the USA. Hospitals participating in the registry submit clinical information regarding the medical history, hospital care, and outcomes of consecutive patients hospitalized for cardiac arrest using an online, interactive case report form and Patient Management Tool™ (IQVIA, Parsippany, New Jersey). QVIA serves as the data collection (through their Patient Management Tool—PMT™) and coordination center for the American Heart Association/American Stroke Association Get With The Guidelines® programs. All participating institutions were required to comply with local regulatory and privacy guidelines and, if required, to secure institutional review board approval. Because data were used primarily at the local site for quality improvement, sites were granted a waiver of informed consent under the common rule. A designation of non-human research was granted by our IRB (Lahey Hospital IRB 20223144).

We included cases between January 2019 and December 2021 to capture the peri-COVID-19 pandemic period. The GWTG-R data collection form includes a variable to identify the application of mCPR device during resuscitation efforts. The mCPR device field is optional in the case report form, and patients without “yes” selected were assumed to have received manual CPR.

An analysis was performed to look at rates of mCPR per month during the study period. To assess changes in mCPR use, we performed logistic regression analysis. Use of mCPR was the dependent variable, and time in 6-month epochs was the primary exposure. The period of January–June 2019 was selected as the reference period as the pandemic largely began March 2020 in the USA. Two specific time periods of focus were January 2020–June 2020 and June 2021–January 2021 since the AHA interim guidance statement was released September 2020.

In a secondary analysis, we assessed the number of institutions that had at least a 5% and 15% increase in mCPR use over the study period. We also assessed hospitals that did not use any mCPR in 2019 but used mCPR in at least 5% of IHCA cases in 2021. In total, 124,426 patients from 433 institutions were included between 2019 and 2021, of whom 5017 (4%) received mCPR. In total, 8512 (6.8%) patients had a COVID-19 diagnosis during their hospitalization—502 (10%) in the mCPR group and 8010 (6.4%) with manual CPR only (*p* < 0.001). In total, 302 of 433 (69.7%) sites did not use any mechanical CPR over the duration of the study period.

The use of mechanical CPR increased from 2.4% of IHCA in January 2019 to 6.0% in December 2021 (*p* < 0.001; Fig. 1a). The use of mCPR increased during each 6-month period that was examined (Fig. 1b). Twenty-eight hospitals (6.5%) had at least a 15% increase in use of mCPR cases from their pre-pandemic baseline. Twenty-seven (6.2%) institutions increased from zero mCPR use in 2019 to at least 5% use in 2021.

**Fig. 1:**
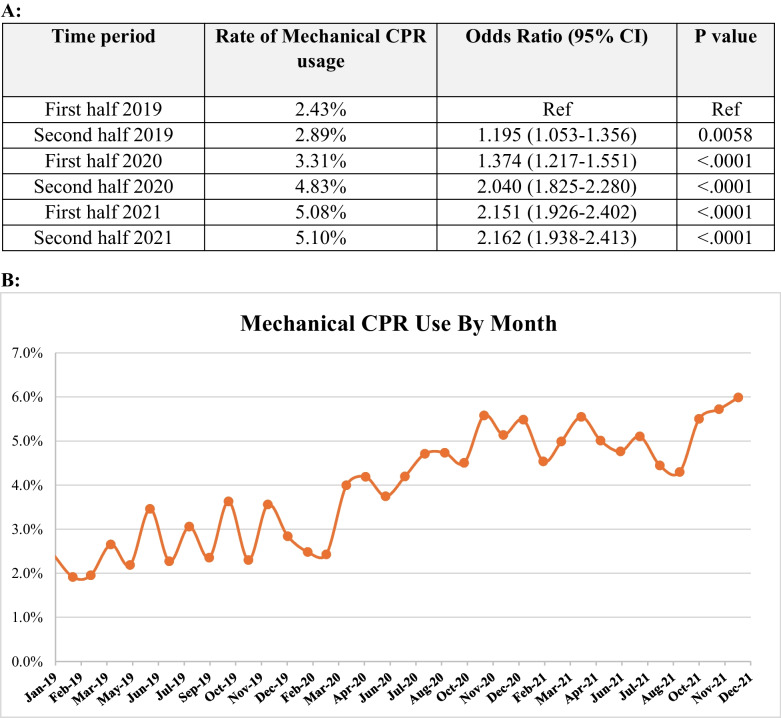
**a** Table demonstrating the rate of change of mCPR in 6-month epochs over the study period. **b** Rate of mCPR per month over the study period

We utilized one of the largest IHCA databases, including over 124,000 patients from 433 institutions. During the study period (January 2019–December 2021), there was an increase in mCPR use from 2.4 to 6.0% during IHCAs, with many institutions using mCPR for the first time. Our findings highlight a substantial increase in mCPR use during the pandemic, including some institutions likely adopting mCPR during the study period. This rapid adoption of mCPR may have been related to the American Heart Association’s recommendations to utilize mechanical CPR in order to protect healthcare staff [1].

As an emergency measure during the pandemic, the AHA made a recommendation for mCPR to protect staff and address staffing limitations. While trials have not demonstrated a clear benefit of mCPR to manual CPR in the out of hospital setting, there is little evidence regarding the use of mCPR for IHCA [3, 4]. Should hospitals consider implementing mCPR we advocate for ongoing assessment of CPR quality and outcomes. Further data are needed to support the use of mCPR for IHCA.

## Data Availability

The datasets used and/or analyzed during the current study are available from the American Heart Association.
